# The Effect of Novel 7-methyl-5-phenyl-pyrazolo[4,3-*e*]tetrazolo[4,5-*b*][1,2,4]triazine Sulfonamide Derivatives on Apoptosis and Autophagy in DLD-1 and HT-29 Colon Cancer Cells

**DOI:** 10.3390/ijms21155221

**Published:** 2020-07-23

**Authors:** Agnieszka Gornowicz, Anna Szymanowska, Mariusz Mojzych, Krzysztof Bielawski, Anna Bielawska

**Affiliations:** 1 Department of Biotechnology, Medical University of Bialystok, 15-222 Bialystok, Poland; anna.szymanowska@umb.edu.pl (A.S.); anna.bielawska@umb.edu.pl (A.B.); 2 Department of Chemistry, Siedlce University of Natural Sciences and Humanities, 08-110 Siedlce, Poland; 3 Department of Synthesis and Technology of Drugs, Medical University of Bialystok, 15-222 Bialystok, Poland; kbiel@umb.edu.pl

**Keywords:** colorectal cancer, apoptosis, autophagy, anticancer agents, roscovitine, 5-fluorouracil

## Abstract

The discovery of cytotoxic drugs is focused on designing a compound structure that directly affects cancer cells without an impact on normal cells. The mechanism of anticancer activity is mainly related with activation of apoptosis. However, recent scientific reports show that autophagy also plays a crucial role in cancer cell progression. Thus, the objective of this study was to synthesize 7-methyl-5-phenyl-pyrazolo[4,3-*e*]tetrazolo[4,5-*b*][1,2,4]triazine utilizing nucleophilic substitution reaction at the position N1. The biological activity of tested compounds was assessed in DLD-1 and HT-29 cell lines. The induction of apoptosis was confirmed by Annexin V binding assay and acridine orange/ethidium bromide staining. The loss of mitochondrial membrane potential and caspase-8 activity was estimated using cytometer flow analysis. The concentration of p53, LC3A, LC3B and beclin-1 was measured using the ELISA technique. Our study revealed that anticancer activity of 7-methyl-5-phenyl-pyrazolo[4,3-*e*]tetrazolo[4,5-*b*][1,2,4]triazine derivatives is related with initiation of apoptosis occur on the intrinsic pathway with mitochondrial membrane decrease and extrinsic with increase of activity of caspase-8. Moreover, a decrease in beclin-1, LC3A, and LC3B were observed in two cell lines after treatment with novel compounds. This study showed that novel 7-methyl-5-phenyl-pyrazolo[4,3-*e*]tetrazolo[4,5-*b*][1,2,4]triazine derivatives might be a potential strategy in colon cancer treatment.

## 1. Introduction

Colorectal cancer (CRC) represents a major health problem and ranks as the second most lethal malignancy worldwide. Surgery, chemotherapy, and targeted therapy are still the most common methods of treatment of patients with CRC. Chemotherapy is based on single agents such as fluoropyrimidine (5-FU) and multiple agent therapy, which includes oxaliplatin, capecitabine, and irinotecan. Unfortunately, some adverse effects and limitations such as increased toxicity, resistance and low selectivity towards cancer cells give the basis to look for novel approaches and new chemotherapeutic agents. Other therapeutic strategies involve antibodies (cetuximab, panitumumab, bevacizumab) against molecular targets such as epidermal growth factor receptor (EGFR) and vascular endothelial growth factor (VEGF) [1]. Research on the development of a cytotoxic drug is aimed at designing a compound structure, whose action is directed at cancer cells while not affecting normal cells.

Aza-heterocyclic structures occupy a special place in the search for new biologically active molecules, including anticancer activity. Among all heterocycles, the triazine scaffold occupies a prominent position, due to a broad range of biological activities. Many mono and condensed 1,2,4-triazines showed promising biological activities, such as anti-inflammatory, anti-mycobacterial, anti-viral, anti-cancer etc., which makes the triazine ring an attractive scaffold for the design and development of new drugs [2,3,4,5,6]. Seven naturally occurring pyrazolo[4,3-*e*][1,2,4]triazines, pseudoiodinine [7], nostocine A [8], and fluviols A-E [9] were found as extracellular metabolites of some microorganism of the class *Pseudomonas fluorescens* var. *pseudoiodinine* and *Nostoc spongiaeforme*. Therefore, the broad spectrum of biological activity of this group attracts attention in the field of medical chemistry. The outstanding development of triazine derivatives in diverse diseases within a very short span of time proves its magnitude for medicinal chemistry research. Therefore, these compounds have been synthesized as target structure by many researchers and were further evaluated for their biological activities [4,10,11,12]. Synthetically obtained derivatives of the pyrazolo[4,3-*e*][1,2,4]triazine ring system have been extensively studied for their antitumor activity [13,14,15] and as inhibitors of tyrosine kinases [16], tyrosinase [17,18], urease [18], phosphodiesterase 5 (PDE5) [17], carbonic anhydrase (CA), and especially its two isoenzymes associated with cancer i.e., CAIX and CAXII [13,19,20]. In the group of condensed pyrazolotriazines, particular attention should be paid to tricyclic derivatives with a terminal tetrazole ring, which show high anti-tumor activity in the nanomolar concentration range [14,21]. The combination of the pyrazolo[4,3-*e*]tetrazolo[4,5-*b*][1,2,4]triazine core with pharmacophore groups enables the design of novel derivatives with potential biological activity. An important pharmacophore group is a sulfonamide moiety characteristic for many chemical compounds used in medicine. Its importance stems from diverse biological activities such as antibacterial, anti-malarial, hypotensive, diuretic, hypoglycemic, anti-thyroid, anti-parasitic, anti-inflammatory, and anti-glaucomatous activity [5,22]. Furthermore, many studies from various scientific centers have shown that sulfonamides may exhibit an antitumor effect. Therefore, sulfonamides have been particularly considered as essential scaffolds for developing new medicines.

The aim of the study was to synthesize and characterize the chemical structure and molecular mechanism of action of novel 7-methyl-5-phenyl-pyrazolo[4,3-*e*]tetrazolo[4,5-*b*][1,2,4]triazine sulfonamide derivatives (MM124 and MM137) in DLD-1 and HT-29 human colorectal cell lines. Anticancer activity was checked by several biological studies, where the viability and proliferation of colorectal cancer cells were analyzed. The influence of the tested agents on the induction of apoptosis was confirmed by Annexin V binding assay and acridine orange/ethidium bromide staining. The loss of mitochondrial membrane potential, caspase-8 activity and concentration of p53 were also tested. Finally, the levels of LC3A, LC3B and beclin-1 were measured to check if the novel compounds could effect autophagy.

## 2. Results

**Scheme 1:**
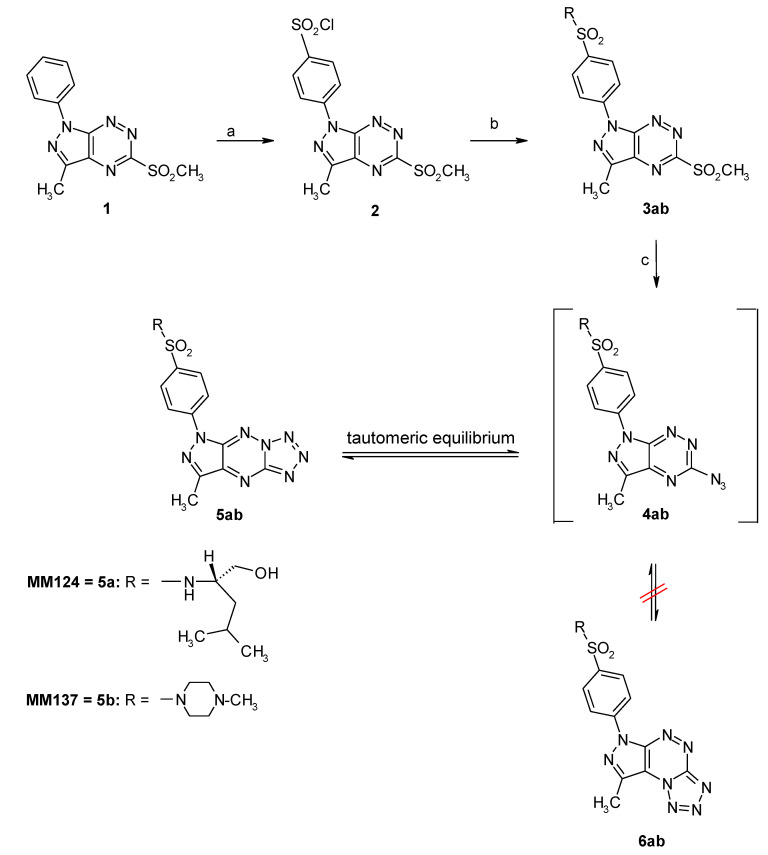
Reagents and conditions. (**a**) ClSO_3_H, 0 °C to rt, 2 h; (**b**) (S)-leucinol or *N*-methyl-piperazine, MeCN, rt, overnight; (**c**) NaN_3_, EtOH, reflux, 18 h.

3-Methyl-5-methylsulfonyl-1-phenyl-1*H*-pyrazolo[4,3-*e*][1,2,4]triazine (**1**) was prepared using a standard procedure described in the literature [23]. Chlorosulfonylation of the pyrazolotriazine **1** in chlorosulfonic acid at 0 °C proceeded smoothly and selectively at the 4′-position of the phenyl ring to give the desired compound **2** in excellent yield. The chlorosulfonyl derivative **2** was readily coupled with (S)-leucinol or *N*-methyl-piperazine in acetonitrile at room temperature to produce the sulfonamides **3ab**, as shown in Scheme 1.

In a further stage of the study, we have attempted the synthesis of 5*H*-pyrazolo[4,3-*e*]tetrazolo[4,5-*b*][1,2,4]triazines with sulfonamide phenyl group at the position N1 (Scheme 1) using nucleophilic substitution reaction of methylsulfonyl group by sodium azide. As a result of these reactions, two new derivatives: **5a (MM124)** and **5b (MM137)** were received and characterized using ^1^H and ^13^C NMR spectroscopy, HRMS, and elemental analysis. The spectral data confirmed that all of the new sulfonamides had the expected structures and were of high purity. At this point, it should also be noted that, in the last stage, i.e., nucleophilic substitution reaction of the methylsulfonyl group with sodium azide, the corresponding derivatives **4ab** were formed as intermediate compounds. Our previous studies using x-ray analysis [24,25] demonstrated that cyclization of the -N_3_ group in N1-substituted 1*H*-pyrazolo[4,3-*e*][1,2,4]triazine goes to the nitrogen atom N6, not to N4 (Scheme 1). Moreover, tautomeric equilibrium between 5-azido-1*H*-pyrazolo[4,3-*e*][1,2,4]triazine and suitable tetrazole derivative we have investigated by ^1^H NMR in deuterated solvents. In all used deuterated solvents (CDCl_3_, DMSO-d_6_, MeOH-d_4_, acetone-d_6_), we observed that tautomeric equilibrium shifted toward tetrazole form. The largest shift of the tautomeric equilibrium was found in acetone because, after 48 h, the concentration of the azide form was almost zero. Detailed research on tautomeric equilibrium in tricyclic sulfonamide derivatives are under study and will be presented in a separate paper.

**Figure 1:**
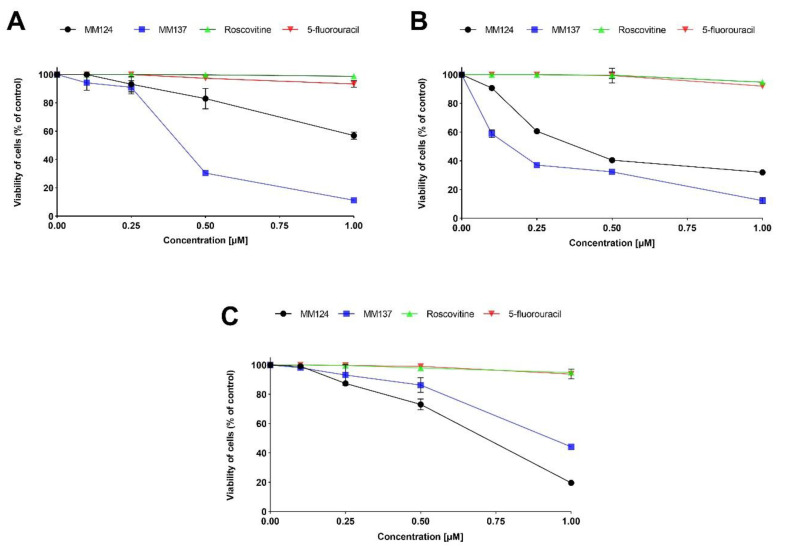
Viability of DLD-1 (**A**), HT-29 (**B**) human colorectal adenocarcinoma cell lines, and human skin fibroblasts (**C**) incubated for 24 h with novel 7-methyl-5-phenyl-pyrazolo[4,3-*e*]-tetrazolo[4,5-*b*][1,2,4]triazine sulfonamide derivatives (MM124, MM137), 5-fluorouracil, and roscovitine. Mean ± SD from three independent experiments done in duplicate are presented.

Novel 7-methyl-5-phenyl-pyrazolo[4,3-*e*]tetrazolo[4,5-*b*][1,2,4]triazine sulfonamide derivatives decrease cell viability and cell proliferation in DLD-1, HT-29, and human skin fibroblasts. The effect of various concentrations of the novel tested compounds (MM124, MM137) and references compounds (5-fluorouracil, roscovitine) on the viability of cell lines was checked by MTT assay (Figure 1).

After 24 h incubation, we demonstrated that MM137 reduced the cell viability in the highest degree with its IC_50_ values 0.43 µM in DLD-1 cells and 0.16 µM in HT-29 cells. IC_50_ values for MM124 were 1.54 µM in DLD-1 cells and 0.41 µM in HT-29 cells. 5-fluorouracil and roscovitine were not so efficient in decreasing the viability of colon cancer cells as newly synthesized compounds and their IC_50_ values were higher than 1 µM in DLD-1cells and higher than 1 µM in HT-29 cells. In addition, the MM124 and MM137 represented slighter cytotoxic activity in human skin fibroblasts than in colon cancer cell lines. The viability of fibroblasts after 24 h exposure to MM124 and MM137 at a concentration 0.5 µM, which was used for further experiments was reduced by 26.92% and 13.72%, respectively.

**Figure 2:**
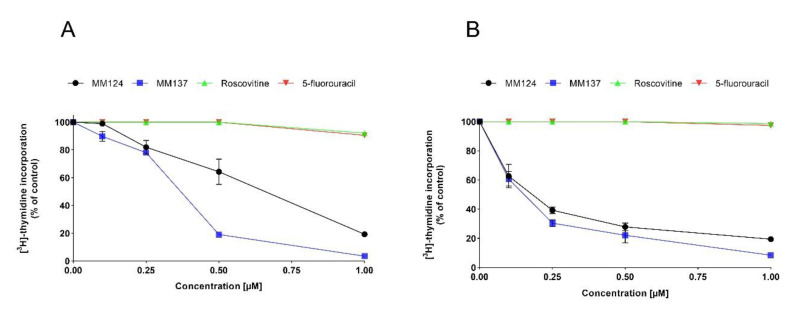
Antiproliferative effects of novel 7-methyl-5-phenyl-pyrazolo[4,3-*e*]tetrazolo[4,5-*b*][1,2,4]triazine sulfonamide derivatives (MM124, MM137), 5-fluorouracil and roscovitine in DLD-1 (**A**) and HT-29 (**B**) cells after 24 h incubation was measured by inhibition of [^3^H]-thymidine incorporation into DNA. Mean ± SD from three independent experiments done in duplicate are presented.

To investigate the effect of MM137, MM124, 5-fluorouracil, and roscovitine on cell proliferation in human colon cancer cells, the incorporation of [^3^H]-thymidine to DNA was analyzed (Figure 2).

The exposure of the cells to the tested compounds inhibited the cell proliferation in a dose-dependent manner. The IC_50_ values for MM137 were 0.39 µM in DLD-1 cells and 0.15 µM in HT-29 cells. MM124 also possessed high antiproliferative potential and its IC_50_ values were 0.74 µM in DLD-1 cells and 0.19 µM in HT-29 cells. The reference compounds did not represent such strong antiproliferative properties like newly synthesized 7-methyl-5-phenyl-pyrazolo[4,3-*e*]tetrazolo[4,5-*b*][1,2,4]triazine sulfonamide derivatives, and their IC_50_ values were higher than 1 µM in DLD-1 and HT-29 cells.

**Figure 3:**
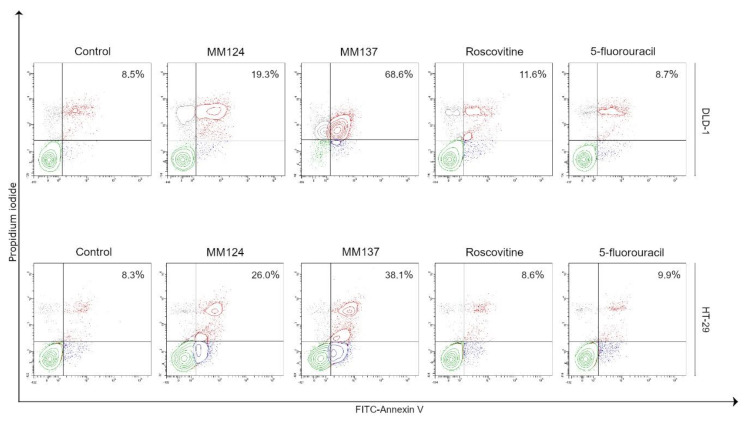
Induction of apoptosis in human HT-29 and DLD-1 cells incubated for 24 h with novel 7-methyl-5-phenyl-pyrazolo[4,3-*e*]tetrazolo[4,5-*b*][1,2,4]triazine sulfonamide derivatives (MM124, MM137), 5-fluorouracil, and roscovitine at 0.5 µM. Concentration was evaluated by Annexin V binding assay. The number of early and late apoptotic cells is presented as mean percentage value from three independent experiments done in duplicate.

Novel 7-methyl-5-phenyl-pyrazolo[4,3-*e*]tetrazolo[4,5-*b*][1,2,4]triazine sulfonamide derivatives induce apoptosis in DLD-1 and HT-29 cells. The results from Annexin-V binding assay are in the agreement with MTT assay (Figure 3). MM137 was the most significant inducer of apoptosis in both analyzed colon cancer cells. We detected 68.6% of early and late apoptotic DLD-1 cells, whereas after 24 h incubation with MM124, we showed 19.3% of apoptotic cells. The weakest pro-apoptotic effect was demonstrated after exposition of cells to roscovitine and 5-fluorouracil, where the percentage of apoptotic cells was 11.6% and 8.7%. The similar pattern was observed in HT-29 colon cancer cells (Figure 3).

The highest number of early and late apoptotic cells (38.1%) in HT-29 was shown after 24 h incubation with MM137. The weaker potential was demonstrated after treatment with MM124, where 26% of HT-29 cells were in apoptosis. Roscovitine and 5-fluorouracil at 0.5 µM concentration did not effect apoptosis. The number of apoptotic cells was similar to the untreated control (8.6% and 9.9%).

**Figure 4:**
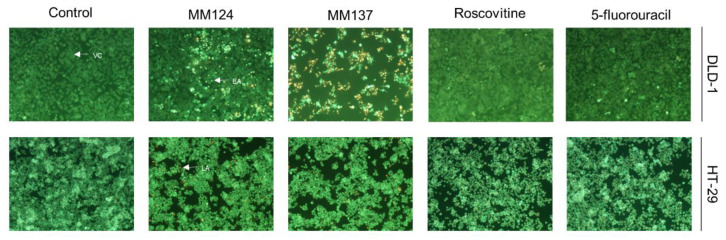
Induction of apoptosis in human HT-29 and DLD-1 cells incubated for 24 h with novel 7-methyl-5-phenyl-pyrazolo[4,3-*e*]tetrazolo[4,5-*b*][1,2,4]triazine sulfonamide derivatives (MM124, MM137), 5-fluorouracil and roscovitine at 0.5 µM, Concentration was evaluated by a fluorescent microscopy after acridine orange and ethidium bromide staining.

In order to investigate and confirm the effect of 7-methyl-5-phenyl-pyrazolo[4,3-*e*]tetrazolo[4,5-*b*][1,2,4]triazine sulfonamide derivatives on the induction of apoptosis, the staining of colon cancer cells with acridine orange/ethidium bromide was performed. The results are presented in Figure 4.

Our studies revealed that MM137 strongly induced programmed cell death, and the highest number of apoptotic cells was observed in comparison with control and other treatments.

**Figure 5:**
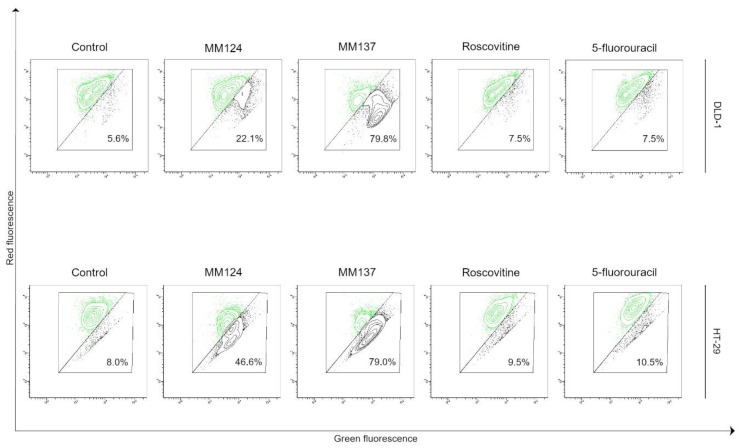
Fluorescence measurement of mitochondrial membrane potential in HT-29 and DLD-1 human colorectal adenocarcinoma cell lines incubated for 24 h with novel 7-methyl-5-phenyl-pyrazolo[4,3-*e*]tetrazolo[4,5-*b*][1,2,4]triazine sulfonamide derivatives (MM124, MM137), 5-fluorouracil, and roscovitine at 0.5 µM concentration. X- and y- axes are green and red fluorescence, respectively. Mean percentage values from three independent experiments done in duplicate are presented.

The loss of mitochondrial membrane potential (MMP) is considered to be an early event in programmed cell death [26]. Studies proved that MM137 decreased the mitochondrial membrane potential in 79.8% of DLD-1 cells and 79% of HT-29 cells, and the difference was statistically significant compared to control (*p* < 0.05). The weaker effect was demonstrated after 24 h incubation with MM124, where 22.1% of DLD-1 cells and 46.6% of HT-29 cells had decreased MMP. Furthermore, our research underlined that roscovitine lowered the MMP only in 7.5% of DLD-1 cells and 9.5% of HT-29 cells. The influence of 5-fluorouracil on MMP in both colon cancer cells was similar to that of roscovitine (Figure 5).

**Figure 6:**
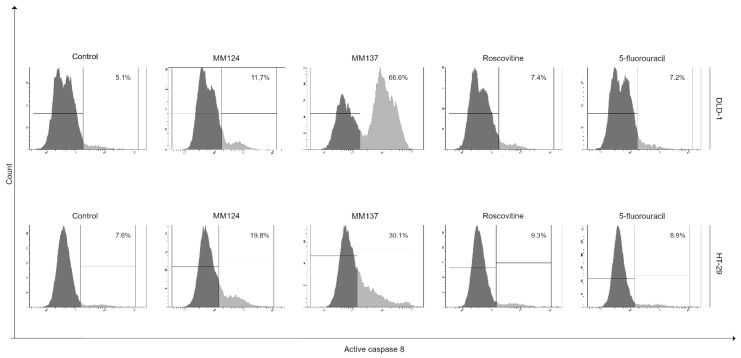
Flow cytometric detection of active caspase-8 in HT-29 and DLD-1 human colorectal cancer cells treated for 24 h with novel 7-methyl-5-phenyl-pyrazolo[4,3-*e*]tetrazolo[4,5-*b*][1,2,4]triazine sulfonamide derivatives (MM124, MM137), 5-fluorouracil, and roscovitine at 0.5 µM concentration. Mean percentage values from three independent experiments done in duplicate are presented.

The activity of caspase-8 was performed to check if our novel derivatives could induce the extrinsic apoptotic pathway. As shown in Figure 6, MM137 was the most effective activator of caspase-8 in colon cancer cells.

It was shown that 66.6% of DLD-1 cells and 30.1% of HT-29 cells had active caspase-8 compared to the control sample. The lesser effect was observed after 24 incubation with MM124. We detected 11.7% of DLD-1 cells and 19.8% of HT-29 cells with active caspase-8. The least significant effect was shown after exposition of cells to reference compounds, such as roscovitine and 5-fluorouracil. In the DLD-1 cell line, we demonstrated 7.4% and 7.2% cells with active caspase-8 after treatment with roscovitine and 5-fluorouracil. In the HT-29 cell line, we noticed 9.3% and 8.9% of cells with active caspase-8. Our studies revealed that only the novel 7-methyl-5-phenyl-pyrazolo[4,3-*e*]tetrazolo-[4,5-*b*][1,2,4]triazine sulfonamide derivatives (MM124 and MM137) were able to induce the extrinsic apoptotic pathway, which was confirmed by the significant increase of the percentage of cells with active caspase-8.

**Figure 7:**
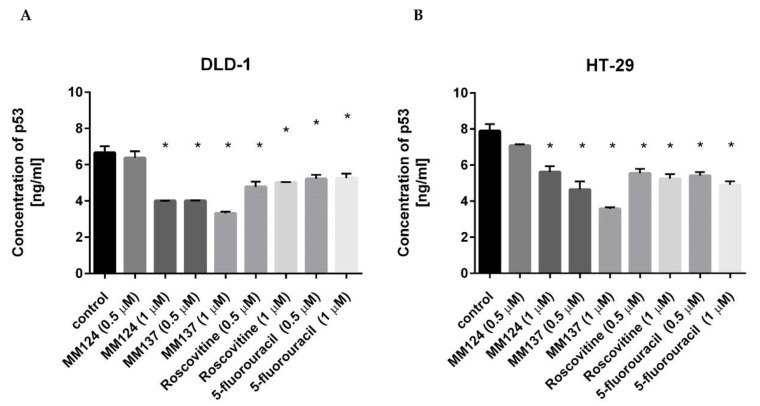
Concentration of p53 in DLD-1 (**A**) and HT-29 (**B**) human colorectal cancer cells after 24 h incubation with novel 7-methyl-5-phenyl-pyrazolo[4,3-*e*]tetrazolo[4,5-*b*][1,2,4]triazine sulfonamide derivatives (MM124, MM137), 5-fluorouracil, and roscovitine. Data is presented as mean ± SD from three independent experiments done in duplicate. * *p* < 0.05 vs. control group.

Novel 7-methyl-5-phenyl-pyrazolo[4,3-*e*]tetrazolo[4,5-*b*][1,2,4]triazine sulfonamide derivatives induce apoptosis in DLD-1 and HT-29 cells independently on p53. We checked whether apoptosis in cancer cells in the presence of test compounds is a p53 protein dependent pathway or not. We checked the concentration of p53 after exposition of the colon cancer cells to all the tested agents. Our studies revealed that all the tested compounds used at concentrations 0.5 µM and 1 µM did not increase the level of p53 (Figure 7).

**Figure 8:**
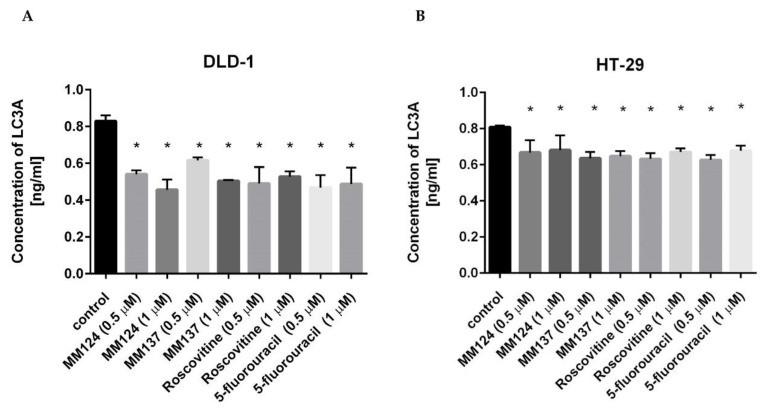
Concentration of LC3A in human DLD-1 (**A**) and HT-29 (**B**) human colorectal cancer cells after 24 h incubation with novel 7-methyl-5-phenyl-pyrazolo[4,3-*e*]tetrazolo[4,5-*b*][1,2,4]triazine sulfonamide derivatives (MM124, MM137), 5-fluorouracil, and roscovitine. Data is presented as mean ± SD from three independent experiments done in duplicate. * *p* < 0.05 vs. control group.

The most significant decrease was observed after 24 h incubation with MM137 in both colon cancer cells. After exposition to the MM137 at two doses 0.5 µM and 1 µM, we detected 4.016 ng/mL and 3.309 ng/mL of p53 in DLD-1 cell lysates in comparison with control (6.66 ng/mL). The concentration of p53 in HT-29 cells was 4.644 ng/mL and 3.581 ng/mL after treatment with MM137 (0.5 µM and 1 µM) compared to the control (7.885 ng/mL). Novel 7-methyl-5-phenyl-pyrazolo[4,3-*e*]tetrazolo[4,5-*b*][1,2,4]triazine sulfonamide derivatives decrease the concentration of LC3A, LC3B, and beclin-1 in DLD-1 and HT-29 cells. Microtubule-associated protein 1A/1B light chain 3A represents an important marker and effector of autophagy [27]. DLD-1 colon cancer cells were exposed for 24 h to the tested compounds at two doses—0.5 µM and 1 µM. We noticed that newly synthesized compounds and the reference compounds led to the decrease of concentration of LC3A. The concentration of LC3A in the untreated cells was 0.83 ng/mL. After exposition of cells to the lower dose of MM137 and MM124, the concentration of LC3A was 0.616 ng/mL and 0.542 ng/mL. The 24 h incubation with roscovitine and 5-fluorouracil (0.5 µM) decreased the concentration of LC3A to 0.49 ng/mL and 0.468 ng/mL. After exposition of cells to the higher dose of MM137 and MM124 (1 µM), the concentration of LC3A was 0.505 ng/mL and 0.456 ng/mL. Twenty-four hours of incubation with roscovitine and 5-fluorouracil (1 µM) decreased the concentration of LC3A to 0.528 ng/mL and 0.488 ng/mL in comparison with the control sample. The similar results were observed in HT-29 colon cancer cells (Figure 8).

The concentration of LC3A in the untreated control was 0.809 ng/mL. All the tested agents reduced the level of protein in cell lysates after 24 h exposition. The differences were statistically significant (*p* < 0.05).

**Figure 9:**
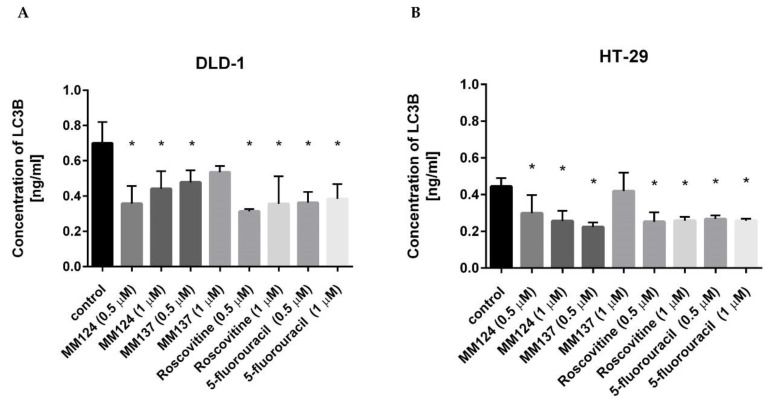
Concentration of LC3B in human DLD-1 (**A**) and HT-29 (**B**) human colorectal cancer cells after 24 h incubation with novel 7-methyl-5-phenyl-pyrazolo[4,3-*e*]tetrazolo[4,5-*b*][1,2,4]triazine sulfonamide derivatives (MM124, MM137), 5-fluorouracil, and roscovitine. Data is presented as mean ± SD from three independent experiments done in duplicate. * *p* < 0.05 vs. control group.

In order to confirm that all the tested agents did not induce autophagy in DLD-1 cells, the microtubule-associated protein 1A/1B light chain 3B levels were checked after 24 h incubation. As demonstrated in Figure 9, LC3B levels were diminished in all cases compared with untreated control samples, respectively.

The concentration of LC3B in the untreated cells was 0.700 ng/mL. After the exposition of colon cancer cells to the lower dose of MM137 and MM124 (0.5 µM), the concentration of LC3B was 0.511 ng/mL and 0.357 ng/mL. The 24 h of incubation with roscovitine and 5-fluorouracil (0.5 µM) decreased the concentration of LC3B to 0.313 ng/mL and 0.362 ng/mL. After exposition of cells to the higher dose of MM137 and MM124 (1 µM), the concentration of LC3B was 0.535 ng/mL and 0.441 ng/mL. The 24 h of incubation with roscovitine and 5-fluorouracil (1 µM) decreased the concentration of LC3B to 0.356 ng/mL and 0.384 ng/mL in comparison with control sample. As revealed in Figure 9, the level of LC3B was also decreased in HT-29 colon cancer cells after 24 h incubation with the tested agents. The concentration of LC3B in the control sample was 0.445 ng/mL. After exposition of colon cancer cells to the lower dose of MM137 and MM124 (0.5 µM), the concentration of LC3B was 0.224 ng/mL and 0.299 ng/mL (*p* < 0.05). Twenty-four hours of incubation with roscovitine and 5-fluorouracil (0.5 µM) decreased the concentration of LC3B to 0.252 ng/mL and 0.267 ng/mL. After exposition of cells to the higher dose of MM137 and MM124 (1 µM), the concentration of LC3B was 0.42 ng/mL and 0.256 ng/mL. Twenty-four hours of incubation with roscovitine and 5-fluorouracil (1 µM) decreased the concentration of LC3B to 0.259 ng/mL and 0.259 ng/mL in comparison with the control sample (*p* < 0.05).

**Figure 10:**
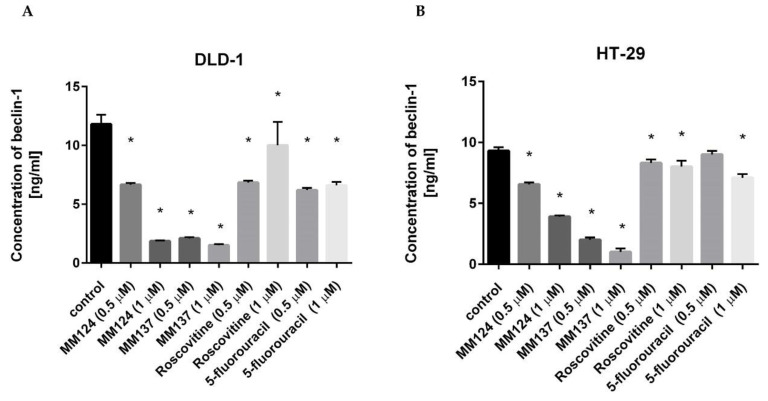
Concentration of beclin-1 in DLD-1 (**A**) and HT-29 (**B**) human colorectal cancer cells after 24 h incubation with novel 7-methyl-5-phenyl-pyrazolo[4,3-*e*]tetrazolo[4,5-*b*][1,2,4]triazine sulfonamide derivatives (MM124, MM137), 5-fluorouracil, and roscovitine. Data is presented as mean ± SD from three independent experiments done in duplicate. * *p* < 0.05 vs. control group.

Additionally, the concentration of beclin-1 was also checked after treatment with the novel and reference compounds. The results showed that MM124 and MM137 more efficiently reduced the concentration of analyzed protein than roscovitine and 5-fluorouracil and the efficiency was enhanced by the higher dose of drug. After exposition to the MM124 at two doses 0.5 and 1 µM we detected 6.65 ng/mL and 1.86 ng/mL of beclin-1 in DLD-1 cell lysates. After 24 h incubation of the cells with MM137, we noticed 2.1 ng/mL and 1.5 ng/mL, respectively. The increasing tendency was observed after 24 h incubation with roscovitine and 5-fluorouracil, but the level did not exceed the value of the control sample (11.8 ng/mL). We detected 6.85 ng/mL and 10 ng/mL after treatment with roscovitine at two concentrations (0.5 µM and 1 µM) as well as 6.2 ng/mL and 6.6 ng/mL of beclin-1 after treatment with 5-fluorouracil. A similar effect was observed in HT-29 cell line, where all the tested compounds also led to the decrease of concentration of beclin-1. However, the strongest inhibitor was MM137, where we detected 2 ng/mL and 1 ng/mL of beclin-1 after exposition to the lower and higher dose of the compound in comparison with control (9.3 ng/mL). After treatment with roscovitine, we revealed 8.3 ng/mL and 8 ng/mL of beclin-1 in cell lysates, whereas we noticed 9 ng/mL and 7.1 ng/mL of beclin-1 after exposition to 5-fluorouracil (Figure 10).

## 3. Discussion

A number of reports showed that 1,2,4-triazines derivatives possess significant antitumor activity and some of them are evaluated under clinical trials [6]. Our studies revealed that both novel 7-methyl-5-phenyl-pyrazolo[4,3-*e*]tetrazolo[4,5-*b*][1,2,4]triazine sulfonamide derivatives MM124 and MM137 induced apoptosis in HT-29 and DLD-1 colorectal cancer cells. The apoptosis is triggered by the activation of caspase-8 and decrease of mitochondrial membrane potential, but the most significant effect of treatment was observed in DLD-1 cells, which are more sensitive to chemotherapy than HT-29 cells [28]. All tested compounds decreased the concentration of p53 in DLD-1 and HT-29 colorectal cancer cells. The above presented facts can be confirmed by numerous literature reports from various scientific centers around the world, as well as our previous publications showing that 1,2,4-triazine is still a valuable source for the design of new biologically active molecules with pyrazolo[4,3-*e*][1,2,4]triazine core [4,10,11,12]. Our earlier studies have shown that derivatives of this system, which has been underrated so far, have the ability to inhibit various enzymes such as tyrosinase, urease, PDE5, and CDK [16,17,18]. In addition, selected pyrazolo[4,3-*e*][1,2,4]triazine sulfonamides proved to be good inhibitors of Bcr-Abl tyrosine-kinase [16] as well as inhibited the two carbonic anhydrase isoenzymes CAIX and CAXII that are associated with cancer [13,19,20].

The last 10 years of research focused on the role of autophagy in carcinogenesis [29]. It was proved that the machinery of autophagy is responsible for changing the microenvironment of tumor cells. The pro-oncogenic factors are released such as pro-inflammatory molecules and/or pro-angiogenic molecules, and their role is based on the support of the tumor growth and progression [30]. Autophagy may be responsible for resistance of cancer cells to many chemotherapeutic agents (i.e., trastuzumab, paclitaxel, epirubicin) [31], and it can be seen as an attractive target for the treatment of patients with colorectal cancer, where almost 20% of them develop metastases. Researchers are constantly looking for autophagy inhibitors that can support the therapeutic effect. Current clinical trials involving autophagy are mainly based on prostate and breast cancer, so there is a rationale for development of novel drugs, which will be able to target autophagy in colorectal cancer [32]. Hydroxychloroquine (HCQ) alone and in combination with chemotherapeutic agents (e.g., oxaliplatin, fluoropyrimidines) is tested. Additionally, the studies are based also on combination of hydroxychloroquine and bevacizumab in metastatic CRC (mCRC) patients. The promising results were observed after treatment of mCRC patients with hydroxychloroquine and vorinostat [33]. The randomized phase II clinical trial is ongoing, where vorinostat plus hydroxychloroquine versus regorafenib in mCRC patients are tested [32]. It was also proved that genetic silencing of Beclin1, Atg5, Atg 7, and Atg12 led to the sensitization of chemoresistant cancer cells to multiple chemotherapeutics [31]. The resistance of cancer cells to apoptosis is a major problem to achieve the successful results from the treatment. In colorectal cancer cells, some proteins are over-expressed such as epidermal growth factor receptor (EGFR) and P-gp, as well as some pro-apoptotic proteins (Bax and p53) levels are down-regulated, which was shown in SW620 cells [28]. The restoration of apoptotic signals can be done by the usage of BH3 mimetics (venetoclax, navitoclax, ABT-737, apogossypol, apogossypolone, gossypol, maritoclax, obatoclax, sabutoclax), EGFR inhibitors (fatinib, cetuximab, dacomitinib, erlotinib, gefitinib, lapatinib, matuzumab, neratinib, nimotuzumab, panitumumab, zalutumumab), or agents that can induce p-53 independent apoptosis (triptolide, resveratrol and dihydrotanshinone) [34,35,36,37,38,39]. The results from our study showed that novel compounds had ability to decrease the concentration of beclin-1, LC3A and LC3B in both analyzed colorectal cancer cell lines. The inhibitory rate was stronger than that of 5-fluorouracil and roscovitine, which were used as reference drugs. 5-fluorouracil is a commonly used chemotherapeutic agent in the treatment of colorectal cancer and also in the treatment of stomach, pancreatic, and breast cancer [1]. Its anticancer activity is associated with the inhibition of thymidylate synthase, therefore DNA synthesis and induction of apoptosis [40]. Roscovitine is a promising drug, which is undergoing phase II clinical trials against non-small cell lung cancer and nasopharyngeal cancer and is evaluated as a potential drug in many other diseases such as viral infections, neurodegenerative diseases [41]. It was proven that mechanism of action is associated with the G_1_ and G_2_/M cell cycle arrest and with the inhibition of cyclin-dependent kinase 1 (CDK1) and cyclin-dependent kinase 2 (CDK2). The combination of roscovitine with doxorubicin exerted synergistic cytotoxicity against three cancer cell lines [42,43].

Our study revealed that novel 5-phenyl-7-methyl-pyrazolo[4,3-*e*]tetrazolo[4,5-*b*][1,2,4]triazine derivatives possess promising anticancer properties and represent potential strategy in colon cancer treatment, but further *in vivo* examination is also required.

## 4. Materials and Methods

### 4.1. Synthesis of MM124 and MM137

General melting points were determined in open capillaries and are uncorrected. ^1^H and ^13^C NMR spectra were recorded on a Varian spectrometer (400 MHz for ^1^H and 100 MHz for ^13^C, Siedlce, Poland). The chemical shift values are expressed in ppm (part per million) with tetramethylsilane (TMS) as internal reference. The relative integrals of peak areas agreed with those expected for the assigned structures. Molecular weight of final compounds was assessed by electrospray ionization mass spectrometry (ESI/MS) on an Agilent Technologies 6538 UHD Accurate Mass Q-TOF LC/MS (Agilent Technologies, Inc., Santa Clara, CA, USA). Elemental compositions are within ± 0.4% of the calculated values. Starting compound **1** was synthesized according to the procedure from the literature [23].

#### 4.1.1. General Method Synthesis of Derivative **2**

Compound **1** (1.156 g, 4 mmoL) was added portion wise to stirred and cooled chlorosulfonic acid (2 mL) in an ice bath; the reaction mixture was then warmed to room temperature gradually and stirred for 2 h after the addition. The reaction solution was cautiously added to ice-water (15 mL), and the aqueous mixture was extracted with dichloromethane (4 × 50 mL). The combined extracts were dried over anhydrous Na_2_SO_4_ and evaporated under vacuum to give the required crude sulfonyl chloride (**2**). After evaporation of the solvent, the crude product was purified by column chromatography using methylene chloride/methanol (50:1) as the eluent to produce the product as a yellow solid.

1-[(para-chlorosulfonyl)phenyl]-3-methyl-5-methylsulfonyl-1H-pyrazolo[4,3-e][1,2,4]triazine (**2**):

Yield 65%. Melting point: 173–175 °C; ^1^H NMR (400 MHz, CDCl_3_) δ: ^1^H NMR (CDCl_3_) δ: 2.90 (s, 3H), 3.62 (s, 3H), 8.29 (d, 2H, J = 9.2 Hz), 8.81 (d, 2H, J = 9.2 Hz); ^13^C NMR (CDCl_3_) δ: 11.52, 67.40, 120.15, 129.03, 129.65, 138.01, 142.08, 142.71, 147.33, 148.38, 162.28. HRMS (ESI, m/z) Calcd for C_12_H_10_ClN_5_O_4_S_2_ [M+] 387.8256. Found [M+] 387.8260. Anal. Calcd for C_12_H_10_ClN_5_O_4_S_2_: C, 45.72; H, 3.84; N, 19.04. Found: C, 45.60; H, 3.90; N, 18.90.

#### 4.1.2. Synthesis of Sulfonamides **3ab**

Derivative **2** (194 mg, 0.5 mmoL) was dissolved in anhydrous acetonitrile (5 mL) and appropriate amine (1.75 mmoL) was added. The reaction was stirred overnight at room temperature, and then the reaction mixture was concentrated in vacuo to afford the crude sulfonamide, as a yellow solid. The residue was purified on silica gel using a mixture of CH_2_Cl_2_:EtOH (25:1) as eluent to give the titled compounds as a yellow solid.

*N-(S)-(1-hydroxy-4-methylpentan-2-yl)-4-(3-methyl-5-methylsulfonyl-1H-pyrazolo[4,3-e]*[1,2,4]*triazyn-1-yl)benzenesulfonamide *(**3a**): Yield 94%. Melting point: 132–134 °C; ^1^H NMR (methanol) δ: 0.67 (d, 3H, J = 6.8 Hz), 0.82 (d, 3H, J = 6.8 Hz), 1.25–1.40 (m, 2H), 1.51–1.59 (m, 1H), 2.84 (s, 3H), 3.31–3.35 (m, 2H), 3.43–3.48 (m, 1H), 2.97 (s, 3H), 8.10 (d, 2H, J = 6.8 Hz), 8.62 (d, 2H, J = 6.8 Hz); ^13^C NMR (methanol) δ: 11.15, 22.06, 23.62, 25.36, 41.07, 42.05, 54.93, 65.95, 121.27, 129.62, 139.33, 141.65, 142.20, 147.77, 149.84, 162.83. HRMS (ESI, m/z) Calcd for C_18_H_24_N_6_O_5_S_2_ [M+] 469.1322. Found [M+] 469.1322. Anal. Calcd for C_18_H_24_N_6_O_5_S_2_: C, 46.14; H, 5.16; N, 17.94. Found: C, 46.10; H, 5.35; N, 17.77.

*N-(4-methylpiperazin-1-yl)-4-(3-methyl-5-methylsulfonyl-1H-pyrazolo[4,3-e]*[1,2,4]*triazyn-1-yl)-benzenesulfonamide* (**3b**): Yield 99%. Melting point: 140–142 °C; ^1^H NMR (CDCl_3_) δ: 2.28 (s, 3H), 2.51 (t, 4H, J = 4.8 Hz), 2.89 (s, 3H), 3.11 (bs, 4H), 3.61 (s, 3H), 7.96 (d, 2H, J = 8.8 Hz), 8.64 (d, 2H, J = 8.8 Hz); ^13^C NMR (CDCl_3_) δ: 11.47, 40.76, 45.65, 45.90, 53.94, 119.95, 129.49, 133.94, 137.48, 141.03, 146.59, 148.03, 161.93. HRMS (ESI, m/z) Calcd for C_17_H_21_N_7_O_4_S_2_ [M+] 452.1171. Found [M+] 452.1171. Anal. Calcd for C_17_H_21_N_7_O_4_S_2_: C, 45.22; H, 4.69; N, 21.71. Found: C, 45.17; H, 4.81; N, 21.59.

#### 4.1.3. Synthesis of Tricyclic Sulfonamides

To a solution of sulfonamide derivative with a methylsulfonyl group (**3a** or **3b**, 0.33 mmoL) in anhydrous ethanol (5 mL), sodium azide (21 mg, 0.33 mmoL) was added. The reaction mixture was refluxed until the substrate disappeared (control TLC). Then, the solvent was evaporated, and the crude product was purified using a column chromatography and CH_2_Cl_2_: MeOH (50:1) mixture as eluent to give the final compounds as a yellow solid.

*N-(S)-(1-hydroxy-4-methylpentan-2-yl)-4-[7-methyl-5H-pyrazolo[4,3-e]tetrazolo[4,5-b][1,2,4]triazin-5-yl]-benzenesulfonamide* (**5a, MM124**): Yield 67%. Melting point: 157–160 °C; ^1^H NMR (methanol) δ: 0.66 (d, 3H, J = 6.8 Hz), 0.80 (d, 3H, J = 6.8 Hz), 1.30–1.39 (m, 4H), 1.47–1.57 (m, 1H), 2.85 (s, 3H), 3.24–3.29 (m, 1H), 3.33–3.35 (m, 1H), 3.43 (dd, 1H, J_1_ = 10.8 Hz, J_2_ = 4.4 Hz), 8.08 (d, 2H, J = 8.8 Hz), 8.42 (d, 2H, J = 8.8 Hz); ^13^C NMR (methanol) δ: 11.19, 22.05, 23.62, 25.34, 42.02, 54.88, 65.95, 120.13, 129.69, 140.88, 141.86, 143.30, 148.33, 148.88, 149.31; HRMS (ESI, m/z) Calcd for C_17_H_21_N_9_O_3_S [M+] 432.1561. Found [M+] 432.1561. Anal. Calcd for C_17_H_21_N_9_O_3_S: C, 47.32; H, 4.91; N, 29.22. Found: C, 47.48; H, 5.02; N, 29.10.

*N-(4-methylpiperazin-1-yl)-4-[7-methyl-5H-pyrazolo[4,3-e]tetrazolo[4,5-b]*[1,2,4]*triazin-5-yl]-benzenesulfonamide* (**5b, MM137**): Yield 90%. Melting point: 210–212 °C; ^1^H NMR (CDCl_3_) δ: 2.29 (s, 3H), 2.50 (t, 4H, J = 4.8 Hz), 3.15 (bs, 4H), 3.61 (s, 3H), 7.94 (d, 2H, J = 8.8 Hz), 8.42 (d, 2H, J = 8.8 Hz); ^13^C NMR (CDCl_3_) δ: 11.49, 40.96, 45.55, 46.20, 53.96, 119.95, 129.49, 133.94, 137.58, 141.53, 146.39, 148.23, 149.93. HRMS (ESI, m/z) Calcd for C_16_H_18_N_10_O_2_S [M+] 415.1408. Found [M+] 415.1402. Anal. Calcd for C_16_H_18_N_10_O_2_S: C, 46.37; H, 4.38; N, 33.80. Found: C, 46.42; H, 4.50; N, 33.66.

### 4.2. Cell Culture of HT-29 and DLD-1 Cells

Cell culture HT-29 (HTB-38), DLD-1 (CCL-221) human colorectal adenocarcinoma cell lines and fibroblasts skin cells were acquired from the American Type Culture Collection (ATCC). First cell line was grown in McCoy’s 5A medium (Pan Biotech, Aidenbach, Lower Bavaria, Germany), the second cell line was maintained in RPMI 1640 medium (ATCC, Manassas, VA, USA), and the third one in DMEM (Corning, Kennebunk, ME, USA). The growth supplement for cell culture (fetal bovine serum (FBS—Eurx, Gdansk, Poland)) and antimicrobial substances (penicillin/streptomycin)(Corning, Kennebunk, ME, USA) were added in 10% and 1% concentration, respectively. The incubator asserted appropriate growth conditions which are required for this cell lines: 5% of carbon dioxide, 37 Celsius degree and the humidity between 90% and 95%. The 100 mm plates were used to culture the cells. After a cell line reached about 80–90% confluency, the detachment of cells with 0.05% trypsin containing 0.02% EDTA (Corning, Kennebunk, ME, USA) and PBS (Corning, Kennebunk, ME, USA) was performed. The cells were then reseeded at density 5 × 10^5^ cells per well in six well plate in 1 mL of appropriate medium and after 24 h incubation used in the presented tests.

### 4.3. Cell Viability Assay

The cytotoxic effect of MM124 and MM137 on three cell lines was performed by MTT assay. Roscovitine (Sigma-Aldrich, St Louis, MO, USA) and 5-flurouracil (Sigma-Aldrich, St Louis, MO, USA) were used as reference drugs. The cells were incubated with serial dilution of tested compounds and reference drugs for 24 h in duplicates. Then, the liquid above the cells was removed by aspiration and the cells were three times rinsed with phosphate-buffered saline without calcium and magnesium at room temperature. Afterwards 50 µL of 5 mg per mL of MTT (Sigma-Aldrich, St Louis, MO, USA) was added to 1 mL of PBS. After the required time elapsed the MTT solution was removed and the formazan crystals were dissolved in 1 mL of DMSO (Sigma-Aldrich, St Louis, MO, USA). The 570 nm wavelength was used to measure the absorbance with Spectrophotometer UV-VIS Helios Gamma (Unicam/ThermoFisher Scientific Inc., Waltham, MA, USA). The obtained absorbance in control cells (without compound) was taken as 100%, while the survival of the cells incubated with tested compounds was presents as a percentage of control value [44].

### 4.4. [^3^H]Thymidine Incorporation Assay

The antiproliferative effect of novel synthesized compounds was performed by [^3^H]thymidine incorporation assay as described in the literature [2]. The cells were exposed on various concentrations of 7-methyl-5-phenyl-pyrazolo[4,3-*e*]tetrazolo[4,5-*b*][1,2,4]triazine sulfonamide derivatives and reference drugs for 24 h. After following the incubation, the cells were washed with Phosphate-Buffered Saline (PBS) without Calcium and Magnesium and the fresh medium was added. Then 0.5 µCi of radioactive [^3^H]thymidine was appended and continue the incubation for four hours. In the first step the liquid was expelled and the cells were placed on ice and washed two times with 1 mL of 0.05 M Tris-HCl buffer comprising 0.11 NaCl and two times with 1 mL of 5% TCA acid. Finally, the cells were solubilized at room temperature with 1 mL of 0.1 M NaOH with 1% SDS. Received cell lysates were transmitted to scintillation vials and filled with 2 mL of scintillation fluid. The radioactivity was estimated by liquid scintillation counting on Scintillation Counter 1900 TR, TRI-CARB (Packard, Perkin Elmer, Inc., San Jose, CA, USA). The intensity of DNA biosynthesis in control cells was expressed in dpm of radioactive thymidine incorporated in the DNA of the selected cell lines and was taken as 100%. Values from the tested compounds were expressed as a percentage of the control value [45].

### 4.5. Flow Cytometry Assessment of Annexin V Binding

Flow cytometry assessment of Annexin V binding Apoptosis Detection Kit II (BD Biosciences, San Diego, CA, USA) was utilized to examine the induction of apoptosis in DLD-1 and HT-29 colorectal adenocarcinoma cell lines. The procedure of detection was carried out as maintained by manufacturer’s guidance. Tested compounds were added in 0.5 µM concentration. The experiment was realized exploited utilizing flow cytometer (BD FACSCanto II, Becton Dickinson Biosciences Systems, San Jose, CA, USA). Then received results were investigated with FACSDiva software (ver. 6.1.3, BD Biosciences Systems, San Jose, CA, USA). The assay was performed as described in the literature [46].

### 4.6. Acridine Orange/Ethidium Bromide Fluorescent Staining

The method uses changes in the functioning of the cytoplasmic membrane undergoing apoptosis or necrosis. The modification in permeability of cell membrane permits penetration of fluorescent markers and specific staining viable, dead and apoptotic cells. DLD-1 and HT-29 cells were incubated with tested compounds at 0.5 µM concentration for 24 h. Cells were then stained with 10 µL of a acridine orange and ethidium bromide incubated in room temperature for 5 min. Afterwards cells were examined in a fluorescence microscope Nikon Eclipse Ti connected an inverted camera Nikon Instruments Inc., Melville, NY, USA) using 100× magnification. The results were parsed with NIS-Elements software (ver. 3.00, Nikon Instruments Inc., Melville, NY, USA) [47].

### 4.7. Analysis of Mitochondrial Membrane Potential

Analysis of mitochondrial membrane potential. To determine the changes in mitochondrial membrane potential (ΔΨ), the JC-1 MitoScreen kit (BD Biosciences, San Diego, CA, USA) was utilized. The assay was executed with flow cytometer (BD FACSCanto II, Becton Dickinson Biosciences Systems, San Jose, CA, USA). Novel synthesized compounds and reference compounds were used at 0.5 µM concentration. After 24 h incubation, the assay was performed as described in the literature [26]. The obtained results were analyzed utilizing BD FacsDiva software (ver. 6.1.3, BD Biosciences Systems, San Jose, CA, USA).

### 4.8. Analysis of Caspase-8 Enzymatic Activity

Detection of caspase-8 activity was estimated with FLICA Caspase-8 Assay Kit (ImmunoChemistry Technologies, Bloomington, MN, USA). Both colon adenocarcinoma cell lines (DLD-1 and HT-29) were incubated for 24 h with novel synthesized compounds, roscovitine, and 5-flurouracil at 0.5 µM concentration. The flow cytometer (BD FACSCanto II, Becton Dickinson Bioscences Systems, San Jose, CA, USA) was utilized to measure the percent of activate caspase-8 in both cell lines. Data was acquired and evaluated with FACSDiva software (ver. 6.1.3, BD Biosciences Systems, San Jose, CA, USA). The test was performed as described in the literature [48].

### 4.9. Determination of p53, Beclin-1, LC3A and LC3B

The high sensitivity assay kit (EIAab Science Co., Ltd,Wuhan, China) was used to determine the concentrations of proteins in cell lysates after 24 h of incubation with the tested compounds at 0.5 µM and 1 µM concentrations. Briefly, trypsinized cells were washed three times with cold PBS and centrifuged at 1000× *g* for 5 min at 4 °C. The cells (1.5 × 10^6^) were suspended in lysis buffer for whole cell lysates. After centrifugation the supernatants were frozen immediately at −80 °C.

The microtiter plates provided in this kit have been pre-coated with an antibody specific to analyzed antigen. Standards and samples were incubated for 2 h at 37 °C. Then, the biotin-conjugated antibody was pipetted into the microplate wells. Following a wash step to remove any unbound substances, avidin conjugated to Horseradish Peroxidase (HRP) was added. Then, a substrate solution (TMB) was pippeted and the color developed in the proportion to the amount of antigen in the samples. The addition of a sulfuric acid solution terminated the reaction and the concentration of the antigen was calculated. [49].

## Abbreviations

CRC	colorectal cancer
5-FU	5-fluorouracil
EGFR	epidermal growth factor receptor
VEGF	vascular endothelial growth factor
PDE5	phosphodiesterase 5
CA	carbonic anhydrase
CAIX	carbonic anhydrase IX
CAXII	carbonic anhydrase XII
MMP	mitochondrial membrane potential
LC3A	microtubule-associated protein 1A/1B light chain 3A
LC3B	microtubule-associated protein 1A/1B light chain 3B
ClSO_3_H	chlorosulfonic acid
MeCN	acetonitrile
EtOH	ethanol
HCQ	hydroxychloroquine
mCRC	metastatic colorectal cancer
CDK1	cyclin-dependent kinase 1
CDK2	cyclin-dependent kinase 2
